# Predictive Role of Representations on Interpersonal Life Satisfaction During In Vitro Fertilization Cycles

**DOI:** 10.7759/cureus.91405

**Published:** 2025-09-01

**Authors:** Meropi Moutzouri, George Koulierakis, Antigoni Sarantaki, Paraskevi Giaxi, Panagiota Tzela, Kleanthi Gourounti

**Affiliations:** 1 Department of Midwifery, University of West Attica, Athens, GRC; 2 Laboratory of Epidemiology, Health Determinants, and Well-Being, Department of Public Health Policy, School of Public Health, University of West Attica, Athens, GRC

**Keywords:** cognitive perceptions, common-sense model, dyadic approach, infertile couples, ivf, representations, satisfaction with life

## Abstract

Introduction

Research on representations and their associations with psychological adjustment during the experience of infertility and its treatment has mostly focused on the intrapersonal level. As both partners are involved in and affected by the infertility and its treatment, the aim of the current study was to expand this research and investigate the role of representations on emotional adaptation at an interpersonal level in the light of the Common-Sense Model.

Materials and methods

This cross-sectional study included infertile couples undergoing oocyte retrieval for an in vitro fertilization (IVF) cycle in an assisted reproduction unit in Heraklion, Greece (57 couples) and an infertility center in Athens, Greece (33 couples). Participants filled in the Satisfaction with Life Scale and the Revised Illness Perception Questionnaire (IPQ-R) regarding the timeline, consequences, personal and treatment control over infertility, problem coherence, and emotional representations. The Spearman Rho test was chosen to examine the correlations between the representations of IPQ-R and satisfaction with life of infertile men and women. The predictive role of one partner’s perceptions on the other partner’s positive psychological adaptation was examined through multiple linear regression models.

Results

Correlations were detected between representations of one partner and the life satisfaction of the other partner. During IVF, men’s satisfaction with life was predicted by women’s cognitive representations regarding timeline (Β = 0.200, p=0.009). Women’s satisfaction with life was predicted by men’s perceptions about treatment control over the fertility problem (Β = -0.226, p=0.033).

Conclusion

One partner’s positive psychological adjustment was predicted by his/her partner’s representations of the fertility problem. Examining positive emotions, such as satisfaction with life, is important in order to evaluate the psychological adjustment of infertile couples and to design psychological theory-based interventions to help infertile couples improve their well-being during the stressful experience of IVF cycles.

## Introduction

According to the Common-Sense Model (CSM) [1], patients develop cognitive representations - such as beliefs and perceptions - of a health problem to help them understand and cope with it. These representations, which include perceptions of causes, timeline, controllability, consequences, symptoms, illness coherence, and emotional responses, play a crucial role in shaping patients’ psychological adjustment to the condition. 

The model has been utilized to evaluate the association between beliefs and emotional adaptation in the context of various chronic medical diseases, such as chronic pain [2], psoriasis [3], and various types of cancer [4-5]. There is strong evidence that certain illness perceptions, as outlined by the CSM, can effectively predict the psychological adjustment of patients with various chronic medical conditions [6].

Infertility is not a chronic illness or a life-threatening condition in the precise meaning of these terms, as the infertile couples are generally healthy and do not face any threat to their lives, yet the fertility problem shares common features with chronic illnesses [7]. More specifically, fertility problems often result from anatomical or physiological dysfunctions in the reproductive system. Additionally, in both cases, individuals have to utilize healthcare services. Similar to patients with chronic illnesses, infertile individuals typically deal with the problem over an extended period of time [8]. Moreover, infertile couples often perceive infertility as a state of low control, believing that little or nothing can be done to address it, a perception also common among patients with chronic diseases [8-9]. As far as the consequences, infertility can negatively affect various aspects of life, including social interactions and marital relationships, impacts that are similarly experienced by individuals with chronic illnesses [10].

In addition to the above shared characteristics, infertility, like chronic illness, carries a significant social and emotional burden. Applying the CSM to infertility enables exploration of how both individual and shared experiences between partners contribute to the perception and psychological impact of the condition. This particular theory emphasizes not only internal processes but also external and social factors, such as family and partner dynamics, partner communication, and mutual decision-making, in shaping one’s perceptions and adjustment to infertility [11-12]. This "interpersonal" perspective is particularly relevant in the context of couples undergoing in vitro fertilization (IVF), as both partners are actively involved in the diagnosis, treatment decisions, and outcomes.

The dual nature of infertility, regardless of the diagnosed cause, necessitates joint decision-making throughout the treatment process. Couples navigate the medical complexities together, from initial examinations to critical choices about assisted reproduction technologies [13]. For instance, decisions about initiating an in vitro fertilization (IVF) cycle or determining next steps after an unsuccessful attempt require mutual consideration and agreement. This shared experience underscores the relevance of CSM in understanding the emotional journey of individuals undergoing IVF treatment.

Based on the CSM, research on representations and their associations with emotional adjustment during the experience of infertility has mostly focused on the intrapersonal level [14]. To date, the study by Benyamini et al. [15] is the only one known to have investigated these associations from both personal and interpersonal perspectives in light of CSM, to the best of our knowledge. While their research offered valuable insights, it focused on three specific dimensions of illness representations (timeline, consequences, and controllability) as assessed by the Illness Perception Questionnaire (IPQ) [16]. Notably, the controllability scale in the IPQ combines different aspects of control, which may limit the ability to differentiate between personal and treatment-related control.

The present study seeks to extend this work by incorporating a broader range of illness perception dimensions and by addressing the distinction between types of control. Moreover, participants in the Benyamini et al. [15] study were not involved in the same stage of fertility treatment, potentially influencing the uniformity of the findings. Thus, the aim of the current study was to expand this research by investigating the role of representations of infertile couples undergoing the specific stage of oocyte retrieval on partners’ satisfaction with life and wellbeing at an interpersonal level, in the light of the CSM. Our hypothesis was that partners' perceptions would be associated with their partner’s well-being, highlighting interpersonal influences as proposed in the dyadic application of the CSM.

## Materials and methods

Study design and setting

This study employed a cross-sectional design. Participants were couples experiencing infertility, recruited during the oocyte retrieval phase from the Assisted Reproduction Unit at the University General Hospital of Heraklion (PAGNI), Heraklion, Greece, and the Infertility Center at the Maternity Hospital “Elena Venizelou”, Athens, Greece. Both hospitals provided a comprehensive range of infertility treatments and assisted reproduction methods.

Sample calculation and selection

The sample of the current study was calculated based on the overall number of variables used. Previous analyses revealed that the largest linear regression included seven independent variables. There should be approximately 10 individuals per independent variable entered into the multiple regression model or structural model [17-18]. Thus, a sample size of 80 couples was considered adequate to ensure sufficient statistical power. To account for potential dropouts, we increased the target sample size by an additional 30% (24 couples), resulting in a final recruitment goal of 104 couples. Of the 208 eligible individuals, 28 chose to withdraw from the study, resulting in a final sample of 90 couples (180 individuals) who fully completed the questionnaires.

Participants were recruited using a consecutive sampling method from two fertility centers that differed primarily in their geographic and demographic context - one located in the urban setting of Athens, the capital of Greece, and the other in a smaller, regional area. Data were collected using a self-administered printed questionnaire, completed in person. Each partner filled out the questionnaire independently, without assistance from the researchers, to ensure individual responses. For couples who preferred not to complete the questionnaire on-site, an electronic version was available and could be sent via email. Completion of the questionnaire took approximately 20-25 minutes.

Data collection

The collected demographic and clinical data included participants’ gender, age, and number of prior IVF cycles. Perceptions of infertility and life satisfaction were assessed using the following instruments:

Illness Perception Questionnaire - Revised (IPQ-R)

The Illness Perception Questionnaire - Revised (IPQ-R) [19], the updated version of the scale originally created by Weinman et al. [16], based on CSM, has demonstrated strong psychometric properties across various illness populations [20-22]. The questionnaire provides a numerical evaluation of the different elements of illness representations. For this study, “my illness” was replaced with “my fertility problem” to better suit the context of infertility.

Five subscales were used: 1) Τimeline assessed perceived persistence (acute/chronic) and recurrence (cyclical) of the fertility problem (6 items; α = 0.926; e.g., "The fertility problem will last for a long time").; 2) Consequences measured the perceived impact of the fertility problem (6 items; α = 0.775; e.g., “The fertility problem has major consequences on my life”); 3) Personal control evaluated beliefs about personal control over the fertility problem (6 items; α = 0.846; e.g., “Nothing I do will affect the fertility problem”). Treatment control assessed beliefs about the controllability of the fertility problem through treatment (5 items; α = 0.841; e.g., “My treatment can control the fertility problem”); 4) Illness coherence measured the perceived understanding of the fertility problem (5 items; α = 0.816; e.g., “The fertility problem is a mystery to me”); and 5) Emotional representations explored emotional reactions to the fertility problem (6 items; α = 0.882; e.g., “The fertility problem makes me feel angry”).

Items were rated on a 5-point scale (1 = strongly disagree to 5 = strongly agree). Higher scores in each of the above sub-scales represent stronger representations.

Satisfaction With Life Scale (SWLS)

Satisfaction with Life Scale (SWLS) [23] assesses the overall life satisfaction across three dimensions: conscious, cognitive, and critical assessment of an individual's life. It focuses on global life satisfaction rather than specific life domains (e.g., health, finances), allowing individuals to integrate and weigh these domains as they see fit. The scale comprises five statements rated on a 7-point Likert scale (1 = strongly disagree, 7 = strongly agree: a = 0.860).

Ethical considerations

The study was approved by the Ethics Committees of both participating hospitals (PAGNI: #4342l; “Elena Venizelou”: #19229). Participants received comprehensive information about the study's objectives, potential risks and benefits, and data confidentiality procedures. Written informed consent was obtained from all participants before data collection.

Statistical analysis

The Spearman’s Rho (ρ) correlation coefficient was used to assess the relationship between the variables of infertile men and women. Multiple linear regression analysis was used to examine the relationship between one partner's illness representations (predictor variables) and the other partner's life satisfaction (outcome variable). Analyses were conducted using SPSS version 26 (IBM Corp., Armonk, USA). Statistical significance was set at p <0.05.

## Results

Participants’ characteristics

A total of 180 individuals (90 men, 90 women) undergoing IVF treatment participated in this study. The average age was 40.59 years (SD = 9.07), and the mean number of previous IVF cycles was 1.55 (SD = 2.89).

The mean scores and standard deviations of the revised IPQ (IPQ-R) subscales for both men and women are presented in Table 1. Differences were noted in representations and life satisfaction between men and women. On the timeline subscale, women perceived a shorter duration of infertility experience than men. In terms of consequences, both genders showed minor differences, with women slightly more likely to view fertility problems as less severe. Regarding personal control, men reported a slightly stronger belief that they had no control over the issue. Similarly, in treatment control, men felt more strongly that little could be done to manage infertility. Concerning illness coherence, women had a higher perception of lacking a clear understanding of the fertility problem. In emotional representations, men appeared less concerned about infertility than women. As for life satisfaction, the difference between genders was minimal, with men reporting slightly higher satisfaction levels (Table 1). Overall, the differences between men and women in representations were minor, with some fluctuations, but there were no significant disparities.

**Table 1:** Means and standard deviations of IPQ-R subscales and SWLS SD = standard deviations; IPQ-R = Illness Perception Questionnaire - Revised; SWLS = Satisfaction With Life Scale

Variables	Mean ±SD	
	Men	Women
Timeline	17.89 ± 6.22	18.50 ± 5.65
Consequences	17.11 ± 4.72	17.27 ± 4.47
Personal control over the problem	16.99 ± 5.17	16.52 ± 4.23
Treatment control over the fertility problem	11.84 ± 4.27	10.96 ± 2.82
Illness coherence	10.80 ± 3.87	11.34 ± 3.73
Emotional representations	18.38 ± 5.62	16.12 ± 5.18
Satisfaction with life	25.23 ± 5.73	24.90 ± 5.48

Associations between one partner’s representations and the other partner’s life satisfaction

**Figure 1:**
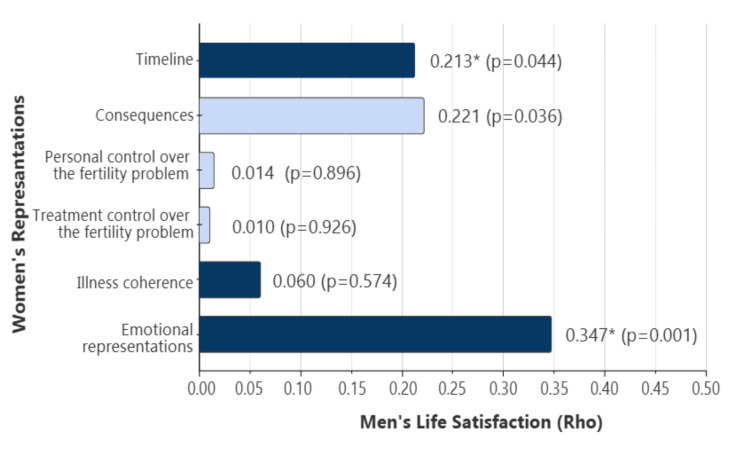
Spearman's rank-order correlation coefficients (ρ) between women’s representations and men’s satisfaction with life ^*^Correlation is significant at the 0.05 level (2-tailed). ^**^Correlation is significant at the 0.01 level (2-tailed). Rho = Spearman’s Rho coefficient; p = statistical significance.

**Figure 2:**
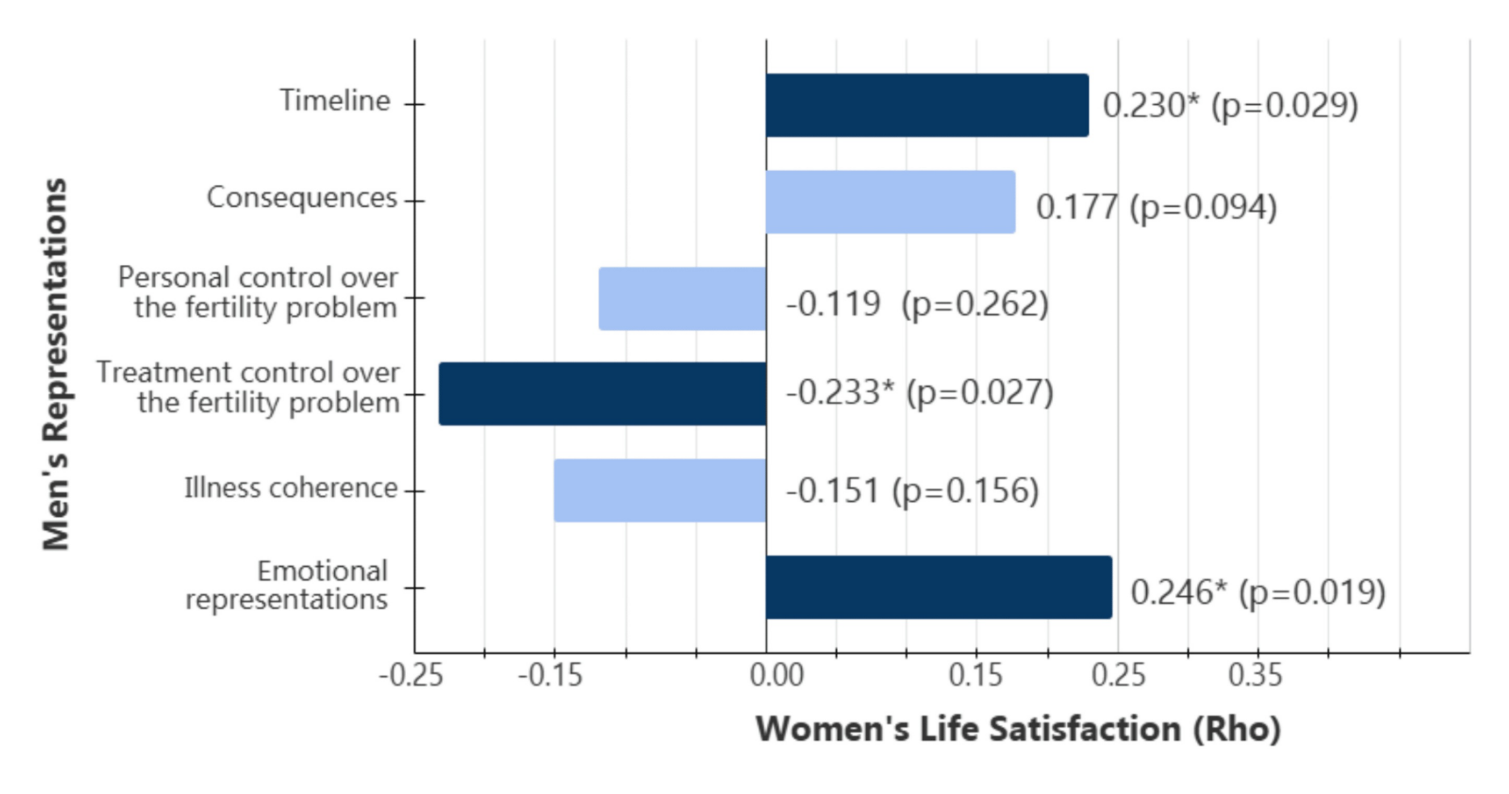
Spearman's rank-order correlation coefficients (ρ) between men’s representations and women’s satisfaction with life ^*^Correlation is significant at the 0.05 level (2-tailed). ^**^Correlation is significant at the 0.01 level (2-tailed). Rho = Spearman’s Rho coefficient; p = statistical significance.

Spearman's Rho correlation coefficients were calculated to examine the associations between one partner’s representations and the other partner’s life satisfaction. The results showed consistent positive correlations between all of the women's representations and men's life satisfaction (Figure 1). In contrast, both positive and negative correlations emerged between men's representations and women's life satisfaction (Figure 2).

Multivariable linear regression models were employed to explore the relationships between one partner’s representations and the other partner’s life satisfaction. Findings revealed that women's perceptions of the IVF treatment timeline were a significant predictor of men's life satisfaction (Β = 0.200, p = 0.009) (Table 2). Conversely, men's perceptions of control over the fertility problem through treatment significantly predicted women's life satisfaction (Β = -0.226, p = 0.033) (Table 2).

**Table 2:** Association between one partner’s representations and other’s partner life satisfaction B = unstandardized coefficients; SE = standard error; β = beta standardized coefficient; t = t-value; p value = statistical significance

Predictor variables	B	SE	β	t	p
Timeline (women)	0.200	0.075	0.209	2.674	0.009
Treatment control over the fertility problem (men)	-0.226	0.104	-0.226	-2.168	0.033

## Discussion

In the descriptive analysis of infertile couples, a gender difference was observed in emotional representations, with men scoring higher than women, suggesting higher concern about fertility issues. Men tended to develop more negative perceptions, focus more on negative information, and ruminate on negative thoughts. An explanation of this result is based on the phase of oocyte retrieval, which is characterised by numerous demands, such as invasive medical procedures, uncertain outcome, lack of control, and lack of active participation of men [24]. Men may feel frustrated since they play a more passive role during this phase, unlike their spouse, who undergoes the medical procedures.

Also, witnessing their partners undergo invasive medical procedures such as oocyte retrieval, potential discomfort, and mood changes due to hormonal treatment can trigger worry in men about women’s health-related risks and the possibility of complications. Moreover, the oocyte retrieval phase is a crucial step, and men may feel fear of failure of the IVF attempt. However, the results of a review indicated that men of the infertile couples reported elevated emotional maladjustment before treatment and after embryo transfer during the time spent waiting for the outcome of the IVF cycle [25]. The difference in the results of the two studies is due to not coherence in terms of their methodology, such as different types of statistical analyses and tools.

The study emphasizes the link between an individual's well-being and their partner’s cognitive representations. This supports Kashy and Kenny’s [11] argument that psychological adjustment to dyadic stressors is shaped not only by one’s own perceptions but also by those of their partner.

The study’s finding regarding the predictive role of women’s cognitive representation of the timeline in men’s life satisfaction should be interpreted in the context of the oocyte retrieval phase of IVF, which is the second-to-last stage of the IVF cycle. At this point, infertility treatment through assisted reproduction is approaching completion. As women are near the end of IVF, they may perceive their infertility journey as more manageable or time-limited. This perception could create a less stressful environment for their partners, contributing to improved well-being in men. Nevertheless, this finding contrasts with Benyamini et al. [15], who reported that an individual’s well-being was associated with their partner’s perception of a longer timeline. This discrepancy may stem from differences in the timing of questionnaire administration between the two studies.

The current study also suggests that men’s belief in the effectiveness of medical treatment for infertility has a negative impact on their partner’s emotional well-being. This counterintuitive association suggests that when men strongly believe the IVF process is effective, this perception may inadvertently place additional expectations or pressure on their partner to achieve a successful outcome. In physically and emotionally demanding stages, such as oocyte retrieval, which can induce significant fear and anxiety in women due to its potential risks and complications [26], such expectations may intensify stress rather than provide reassurance and well-being.

While previous literature indicates that partner support and confidence in treatment can, in some contexts, foster emotional well-being [27], our findings highlight that an overly strong emphasis on medical control might overshadow the emotional coping needs of the partner, thereby diminishing her life satisfaction. A comparison with Benyamini et al. [15] is not feasible due to differences in the methodological tools used in the two studies to assess representations. Specifically, the controllability scale in the IPQ used by Benyamini et al. [15] encompassed all aspects of control without differentiating between personal control and treatment control over the outcome.

Strengths and limitations

The current study is the first and only one to examine the predictive role of all subscales of the IPQ-R on well-being at the interpersonal level during the infertility experience and IVF cycle. A key strength of the present study is its exclusive focus on the egg retrieval phase of the IVF process. Unlike studies that combine participants from multiple treatment stages, our targeted approach minimizes variability related to phase-specific psychological and physiological factors, thereby enhancing the internal validity of the findings.

While the study offers valuable insights, several limitations should be noted: the emotional state of infertile couples before starting assisted reproduction was not assessed, and future research should explore the long-term development of cognitive representations and life satisfaction within this population. Additionally, the sample was recruited by two infertility units during two separate time periods, which may have impacted the results. Although consistent procedures and inclusion criteria were applied, and both clinics followed similar medical protocols and treated comparable populations, potential effects cannot be entirely ruled out across the two fertility centers. Future studies could benefit from collecting data from multiple sites simultaneously to control for potential temporal and contextual variations.

Moreover, a limitation of the present study concerns its non-diachronic cross-sectional design, which does not allow firm causal conclusions to be drawn and is subject to self-report biases, such as recall, social desirability, and subjective interpretation. Future research using longitudinal designs could clarify the directionality of these relationships. Although multiple linear regression analyses were conducted and revealed patterns consistent with potential causal relationships, the temporal precedence of the studied variables cannot be confirmed. In addition, while multiple linear regression is appropriate for the current research scope, more advanced dyadic models, such as the Actor-Partner Interdependence Model (APIM), could further enrich understanding of the interplay between partners’ variables.

## Conclusions

Identifying predictors of life satisfaction, such as a partner’s representations, is vital for professionals in assisted reproduction units. Medical and obstetric personnel need a comprehensive understanding of fertility-related psychological factors to assess and support individuals undergoing IVF effectively. Furthermore, the study underscores the interpersonal nature of infertility and IVF experiences, emphasizing how one partner’s perceptions can affect the other’s psychological adjustment and well-being. Consequently, mental health interventions should be tailored to both individuals and couples, incorporating both individual therapy and couple-based approaches.
